# Hypoxia regulates stemness of breast cancer MDA-MB-231 cells

**DOI:** 10.1007/s12032-016-0755-7

**Published:** 2016-04-01

**Authors:** Jing Xie, Yong Xiao, Xiao-yan Zhu, Zhou-yu Ning, Hai-fan Xu, Hui-min Wu

**Affiliations:** Department of Integrative Oncology, Fudan University Shanghai Cancer Center, Shanghai Medical College, Fudan University, Shanghai, China; Department of Fetal and Neonatal Surgery, Hunan Children’s Hospital, Changsha, Hunan China; Department of Thyroid and Breast Surgery, The First Affiliated Hospital of Nanhua University, Hengyang, 421001 Changsha China; Department of General Surgery, Tongji Hospital, Tongji University School of Medicine, Xincun Road 389, Shanghai, 200065 China

**Keywords:** Breast cancer, Hypoxia, Cancer stem cells, Stem cell niche, Stemness

## Abstract

Human breast cancers include cancer stem cell populations as well as non-tumorigenic cancer cells. Breast cancer stem cells possess self-renewal capability and thus are the root cause of recurrence and metastasis of malignant tumors. Hypoxia is a fundamental pathological feature of solid tumor tissues and exerts a wide range of effects on the biological behavior of cancer cells. However, there is little information on the role of hypoxia in modulating the stemness of breast cancer cells. In the present study, we cultured MDA-MB-231 cells in a hypoxic gas mixture to simulate the hypoxic environment in tissues and to determine how hypoxia conditions could affect the cell proliferation, apoptosis, cytotoxicity, and colony-forming ability. Expression of the stem cell phenotype CD24^−^CD44^+^ESA^+^ was analyzed to assess the effects of hypoxia on stemness transformation in MDA-MB-231 cells. Our results found that the cell toxicity of MDA-MB-231 cells was not affected by hypoxia. Hypoxia could slightly inhibit the growth of MDA-MB-231 cells, but the inhibitory effect is not significant when compared with normoxic control. Moreover, hypoxia significantly blocked the apoptosis in MDA-MB-231 cells (*P* < 0.05). The proportion of CD24^−^CD44^+^ESA^+^ cells in MDA-MB-231 cells was increased greatly after they were treated with hypoxia, and cell colony-formation rate of MDA-MB-231 cells also increased significantly in hypoxia-treated cells. These results encourage the exploration of hypoxia as a mechanism which might not be underestimated in chemo-resistant breast cancer treatment.

## Introduction

Breast cancer is one of the most common malignancies in women and has the highest incidence and mortality rates among all female malignant tumors. According to the World Health Organization, in 2012 there were 1.7 million new breast cancer cases globally and it was responsible for about 0.52 million death cases, representing a significant increase when compared with 2008 statistics [1].

CSCs are the root cause of recurrence and metastasis [2]. Increasing evidence has implicated breast cancer stem cells (BCSCs) as essential for breast cancer development, progression, recurrence, and treatment resistance. Numerous studies have found that CSCs exist in a special microenvironment referred to as their “niche.” The niche can maintain CSCs in a dormant state,ensure their asymmetric division to maintain the proportion of stem cells, and prevent their differentiation [3]. The niche comprises various factors including different types of mesenchymal cells, extracellular matrix, nutrients, temperature, pH, and oxygen concentration. Among these factors, hypoxia is essential to the formation of the CSC niche [4]. Adult stem cell research showed that hypoxia could maintain human embryonic stem cells in an undifferentiated state [5] and bone marrow stem cells in a dormant resting state [6]. Additionally, it previously found a similar effect of hypoxiaon mesenchymal stem cells [7]. In terms of CSCs, hypoxia can enhance cancer cell invasiveness and tumorigenicity as well as strengthening cancer cell stemness [8]. Moreover, hypoxia has been found to facilitate the dedifferentiation of differentiated cancer cells, so as to acquire stemness [9]. The use of anti-angiogenic therapy to treat breast cancer results in ischemic hypoxia in the tumor, which is conducive to the development of BCSCs [10]. Hypoxia can also induce epithelial–mesenchymal transition (EMT) and thus facilitate cancer metastasis [11]. What’s more, the development and metastasis of cancer cells are affected by the hypoxic microenvironment in target organs and tissues [12], and hypoxia can also enhance the resistance of cancer cells to radiotherapy and chemotherapy. These factors contribute to the difficulties in treating cancers [13]. However, there is little information on the role of hypoxia in modulating the stemness of breast cancer cells.

Triple-negative breast cancer (TNBC), which accounts for about 12–17 % of all breast cancer cases, is difficult to treat [14]. The MDA-MB-231 cell line is a representative TNBC cell line, and studies on the biological characteristics and behavior of MDA-MB-231 cells may thus be of great significance. In the present study, we cultured TNBC MDA-MB-231 cells in a hypoxic gas mixture to simulate the hypoxic environment in tissues. The expression of the stem cell phenotype CD24^−^CD44^+^ESA^+^ was analyzed to assess the effects of hypoxia on stemness transformation in MDA-MB-231 cells.

## Materials and methods

### Subjects

The human TNBC MDA-MB-231 cell line was purchased from the Cell Bank of Shanghai Institute of Cell Biology, Chinese Academy of Sciences. The cell culture medium consisted of Leibovitz’s L-15 medium (stored at 4 °C) and fetal bovine serum (FBS) (stored at −20 °C). FBS was thawed before use and mixed with L-15 medium in a 9:1 ratio to prepare the cell culture medium containing 10 % FBS. The prepared medium was dispensed into 50-mL centrifuge tubes and stored at 4 °C before use. Bovine serum albumin (BSA) solution (10 mL) was prepared by dissolving 100 g BSA powder in 10 mL of phosphate-buffered saline. After dissolving thoroughly by stirring, the solution was sterilized by filtering through a 0.22-µm microporous membrane syringe filter to obtain a 1 % BSA solution. BSA solution was prepared freshly before use.

### Recovery and culture of MDA-MB-231 cells

MDA-MB-231 cells were cultured in air using L-15 complete medium containing 10 % FBS, in accordance with the recommendations of the American Type Culture Collection (www.atcc.org/). The air incubator was substituted with a three-gas incubator (ASTEC, Tokyo, Japan), with no external connection to any special gases.

### Establishment of hypoxic cell model

Exponential-phase MDA-MB-231 cells in good condition were collected. Adherent cells were digested with trypsin and counted. The same concentrations of cells were inoculated into cell culture plates or petri dishes with the same specifications for each experiment and then incubated in an incubator. Once the cells established even and fully adherent growth, hypoxia was established using a commercially available modular incubator chamber. The equipment comprised a sealed chamber with one air inlet and one outlet. A petri dish containing sterilized water was placed in the chamber to maintain saturated humidity. According to the experimental design, cells in the hypoxic treatment group were placed in the chamber and aerated continuously with a hypoxic gas mixture (94 % N_2_, 5 % CO_2_, and 1 % O_2_). The oxygen concentration was monitored, and the valve was closed when the oxygen concentration stabilized at 1 %. The air inlet and outlet pipes were clipped sequentially. The cells were cultured under hypoxia at 37 °C, and a normoxic control group was cultured simultaneously in the incubator.

### Cell viability

WST assay (Cell counting kit-8; Dojindo Laboratories, Kumamoto, Japan) was used to detect the effect of hypoxia on MDA-MB-231 cells. Cells were seeded in 96-well plates and labeled in the anoxic treatment group and the normal oxygen group. Cells were digested and diluted to 5 × 10^3^/100 μl and then were found well adhering to the wall after incubation for 2 h. Cells were counted at 2 h, 6 h, 12 h, 24 h, and 48 h using a microtiter-plate colorimetric. All the experiments were conducted in triplicate.

### Cytotoxicity assay

The CytoTox96^®^ Non-Radioactive Cytotoxicity Assay (Promega Co., Madison, WI, USA) was a colorimetric alternative to ^51^Cr release cytotoxicity assays. The assay measured lactate dehydrogenase (LDH), a stable cytosolic enzyme that was released upon cell lysis. Released LDH in culture supernatants was measured with a 30-min coupled enzymatic assay, which resulted in conversion of a tetrazolium salt (INT) into a red formazan product. The amount of color formed was proportional to the number of lysed cells. Visible wavelength absorbance data were collected using a standard 96-well plate reader.

### Apoptosis assay

Annexin V-FITC (Promega Co., Madison, WI, USA) binds with high affinity to PS residues, and PS is exposed during early apoptosis by a flip from the inner to the outer plasma membrane leaflet. Furthermore, PI conjugates to necrotic cells. Cells (5-10x10^4^) were cultured and labeled in the anoxic treatment groups and the normal oxygen groups in their medium. The cells were washed with serum-free medium, and incubation was continued for the desired times. Then, the cells were detached with trypsin solution and washed with FBS. Annexin V-FITC/PI were added into the tube, which was gently mixed at room temperature (20–25 °C) for 10 min in the dark according to the instructions in the manufacturer’s kit. Stained cells were analyzed by using the flow cytometer mentioned above.

### Flow cytometric analysis

Single-cell suspensions obtained from above samples were stained with stem cell markers and analyzed by FACS (BD Biosciences) for their expression of CD24, CD44, and ESA. Data analysis was performed with FlowJo software.

### Statistical analysis

Each experiment was carried out at least three times. All data were expressed as mean ± SEM, where differences were evaluated using Student’s *t* test with the SPSS statistical software program for Microsoft Windows (SPSS Inc., Chicago, IL, USA). A value of *P* < 0.05 was considered statistically significant.

## Results

### Effect of hypoxia on MDA-MB-231 cell proliferation

We tested the effect of hypoxia on MDA-MB-231 cell growth by initially generating a standard curve under normoxic conditions. MDA-MB-231 cells were seeded in culture plates and then observed 1, 1.5, and 2 h later. Cells were almost fully adherent at 2 h after seeding. CCK-8 was added and the optical density (OD_450 nm_) was then measured after 1, 2, and 3 h of reaction. The OD values of replicate wells were averaged, and the average OD value of the background control was subtracted.

**Fig. 1:**
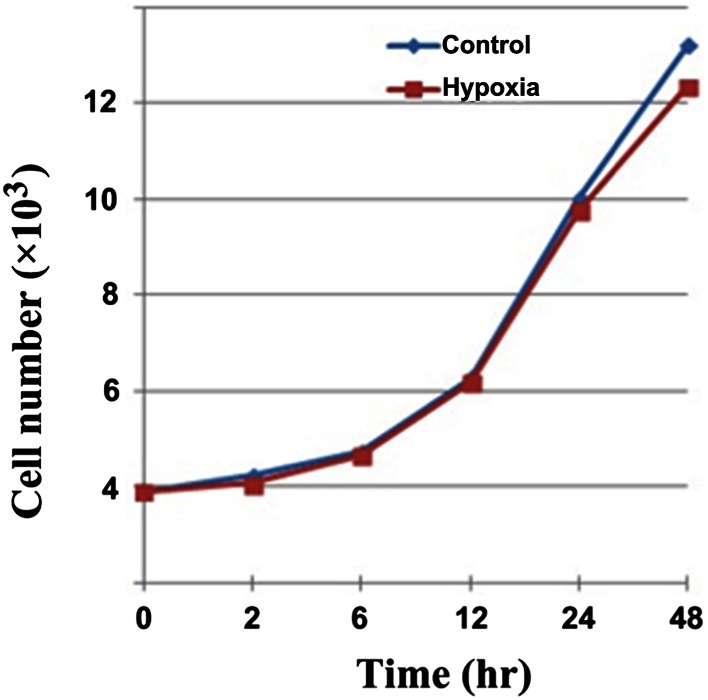
Effect of hypoxia on MDA-MB-231 cell proliferation. The growth curves of the two groups generally overlapped at 2, 6, 12, and 24 h, and the OD values are 0.560 ± 0.026 for control cells and 0.518 ± 0.014 for hypoxic cells (*P* > 0.05)

Based on the standard curve, we chose a cell concentration of 5 × 10^3^ cells/100 μL for seeding the plates. Cells were fully adherent 2 h after seeding. The hypoxia group was then subjected to hypoxic treatment for 48 h, while the control group was treated under normoxic conditions for the same period. We chose 2 h as the best reaction time for CCK-8. The growth curve was plotted with the OD value for the experimental group minus that of the control group on the vertical axis and the time point on the *x*-axis (Fig. 1). The growth curves of the two groups generally overlapped at 2, 6, 12, and 24 h, while separation was most significant at 48 h. The experimental data were analyzed after 48 h, and the OD values were 0.560 ± 0.026 for control cells and 0.518 ± 0.014 for hypoxic cells (*P* > 0.05). Hypoxia resulted in a mild inhibition of cell proliferation, but the inhibitory effect was not significant.

### Cytotoxic effect of hypoxia on MDA-MB-231 cells

**Fig. 2:**
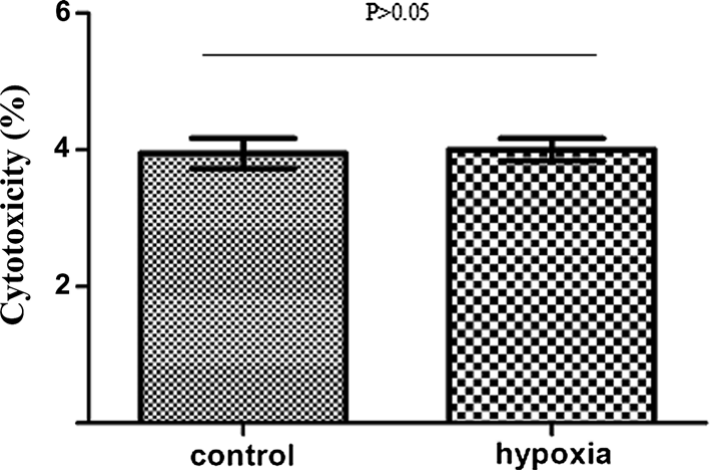
Cytotoxic effect of hypoxia on MDA-MB-231 cells. The cytotoxicity values are 9.871 ± 0.553 % for hypoxic cells and 10.002 ± 0. 417 % for normoxic control cells, which indicated that hypoxic treatment had no significant cytotoxic effect on MDA-MB-231 cells (*P* > 0.05)

MDA-MB-231 cells were divided into hypoxic and normoxic control groups. Hypoxic cells were exposed to 1 % O_2_ for 48 h and then analyzed using the CytoTox 96 non-radioactive cytotoxicity assay kit. The cytotoxicity values were 9.871 ± 0.553 % for hypoxic cells and 10.002 ± 0. 417 % for normoxic control cells (Fig. 2). The results indicated that hypoxic treatment had no significant cytotoxic effect on MDA-MB-231 cells (*P* > 0.05).

### Effect of hypoxiaon MDA-MB-231 cell apoptosis

**Fig. 3:**
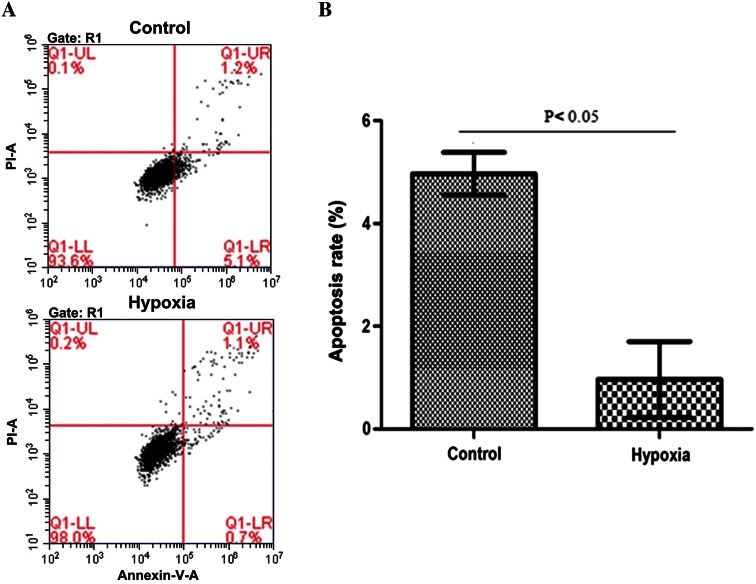
Effect of hypoxia on MDA-MB-231 cell apoptosis. **a** The effect of normoxia and hypoxia on MDA-MB-231 cell apoptosis, and **b** group comparison of apoptosis rate: normoxic control cells showed an apoptotic rate of 4.97 ± 0.42 %, compared with 0.97 ± 0.74 % in the hypoxic cells, indicating that the percentage of apoptotic cells is significantly reduced after 48 h of hypoxia (*P* < 0.05)

MDA-MB-231 cells were divided into hypoxic and normoxic control groups. Hypoxic cells were exposed to 1 % O_2_ for 48 h and then treated with appropriate antibodies before flow cytometry analysis. The effect of hypoxia on MDA-MB-231 cell apoptosis is shown in Fig. 3a. Normoxic control cells showed an apoptotic rate of 4.97 ± 0.42 %, compared with 0.97 ± 0.74 % in the hypoxic cells (Fig. 3b), indicating that the percentage of apoptotic cells was significantly reduced after 48 h of hypoxia (*P* < 0.05).

### Effect of hypoxia on expression of stem cell markers in MDA-MB-231 cells

**Fig. 4:**
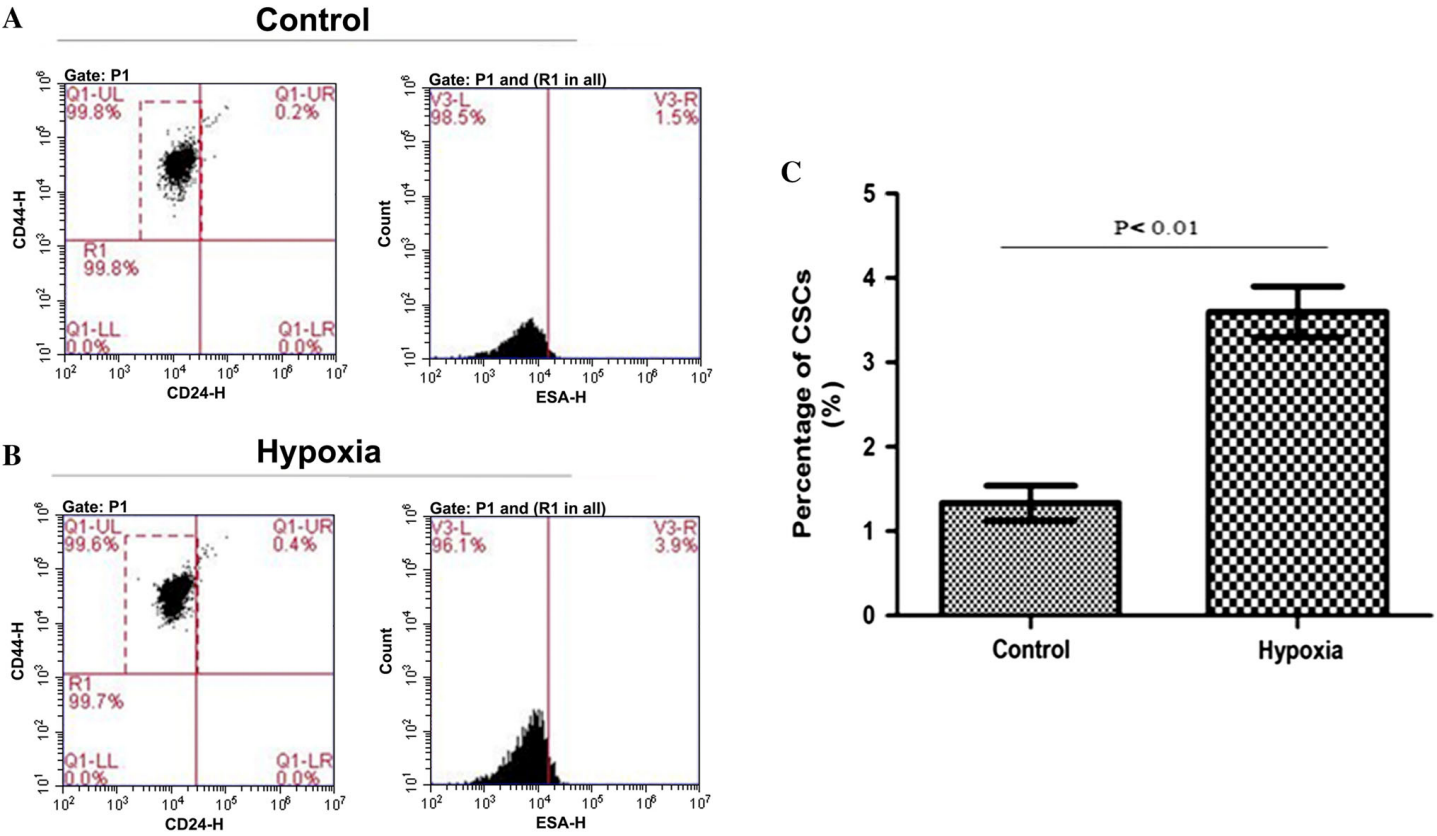
Effect of hypoxia on expression of stem cell markers in MDA-MB-231 cells. **a** Normoxic control group; **b** hypoxic group; and **c** group comparison of percentage of cells expressing CD24^−^CD44^+^ESA^+^: it is significantly higher in the hypoxic group 3.60 ± 0.30 % compared with the normoxic group 1.33 ± 0.21 % (*P* < 0.01)

MDA-MB-231 cells were divided into hypoxic and normoxic control groups. Hypoxic cells were exposed to 1 % O_2_ for 48 h and then incubated with appropriate antibodies before flow cytometry analysis. Cells expressing CD24^−^CD44^+^ESA^+^ in two groups are shown in Fig. 4a, b. The proportion of cells expressing the stem cell marker phenotype was significantly higher in the hypoxic group 3.60 ± 0.30 % compared with the normoxic group 1.33 ± 0.21 %(*P* < 0.01) (Fig. 4c).

### Effect of hypoxia on colony formation in MDA-MB-231 cells

**Fig. 5:**
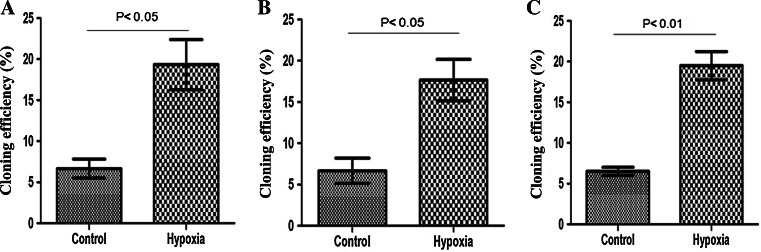
Effect of hypoxia on colony formation by MDA-MB-231 cells. **a**–**c** Show colony-formation rates in MDA-MB-231 cells in the 50- (**a**), 100- (**b**), and 200-cell groups (**c**), respectively

MDA-MB-231 cells were divided into hypoxic and normoxic control groups. Each group of cells was seeded at 50, 100, and 200 cells/dish. Once the cells showed fully adherent growth, the experimental group was exposed to hypoxia for 2 weeks, while the control group was cultured under normoxic conditions for 2 weeks. The numbers of colonies formed in 10-cm petri dishes were counted directly by the naked eye. The colony-formation rate was then calculated using a formula. The normoxic control cells showed a colony-formation rate of 6.67 ± 0.67 % in the 50-cell group, compared with 19.33 ± 1.76 % in the hypoxic group. The equivalent colony-formation rates in the 100-cell groups were 6.67 ± 0.88 % and 17.67 ± 1.45 %, and those in the 200-cell groups were 6.50 ± 0.29 % and 19.50 ± 1.00 % (*P* < 0.05). These results indicated that colony-formation rates of MDA-MB-231 were significantly increased by hypoxia (Fig. 5).

## Discussion

Hypoxia is an important feature of solid tumor tissues and exerts various effects on tumor biology and response to treatment [15]. It has been reported that hypoxia exists in around 50 % of breast cancer cases [16], leading to resistance to chemo-radiotherapy and resulting in treatment failure. The mechanism includes the following several aspects. Hypoxia can alter metabolic pathways and promote cell survival by adaptation to the local microenvironment [17] and can also upregulate the activities of receptor tyrosine kinases and activate multiple signaling pathways conducive to tumor cell proliferation, survival, and metastasis [18]. Furthermore, hypoxia has been shown to induce EMT and facilitate tumor metastasis [19]. However, there were limited existing research reports on the role of hypoxia in modulating the stemness of breast cancer cells.

Normal stem cells are located in a special microenvironment called the “stem cell niche,” comprising immune and mesenchymal stromal cells, vascular networks, various soluble factors, and extracellular matrix components. Niche plays a pivotal role in stem cell proliferation, apoptosis resistance, and stemness regulation. Likewise, the biological behavior of CSCs depends on a CSC niche to maintain their self-renewal capacity, differentiates into the corresponding progenitor cells, and simultaneously remains in an undifferentiated state [20]. Hypoxia is an essential event of the CSC niche [8] and can directly induce self-renewal-associated gene expression and inhibit CSC differentiation. Meanwhile, hypoxia can facilitate the interaction between hypoxia-inducible factors and other signaling pathways to maintain the homeostasis of CSCs and associated metabolic pathways [21]. Tavaluc et al. [16] demonstrated that hypoxia significantly enriched the side population in multiple cell lines. The results of the current study indicated that hypoxia can increase BCSCs in MDA-MB-231 cells, thus providing in vitro evidence for the role of hypoxia in the CSC niche.

The origin of CSCs has long been debated, and two possible origins are currently accepted. First, CSCs may be derived from normal adult stem cells. The second postulated origin of CSCs is associated with EMT. EMT is a characteristic process of morphogenesis that occurs during embryonic development [20, 22]. Morel et al. [20] found that non-tumorigenic mammary epithelial cells can activate the Ras-/mitogen-activated protein kinase signaling pathway to produce CD44^+^CD24^−^ stem-like cells, which exhibit downregulated E-cadherin expression and express the characteristic markers of mesenchymal cells such as vimentin. Mani et al. [23] found that immortalized human mammary epithelial cells underwent EMT and transformed into a stem cell phenotype, with a microsphere-forming ability tenfold higher than that of the control group. In the present study, we verified the regulation of MDA-MB-231 cell stemness by hypoxia by exposing cancer cells to hypoxic conditions and analyzing the quantitative changes in BCSCs by flow cytometry. The results showed that hypoxia induced the expression of stem cell surface markers by MDA-MB-231 cells, suggesting that hypoxia can induce TNBC cells to dedifferentiate and acquire the stem cell phenotype. This dedifferentiation process may be an important origin of BCSCs.

Apoptosis is a pathophysiological process that scavenges useless or harmful cells in the body under normal conditions. It plays an essential role in regulating body development and internal environment homeostasis. Erler et al. [24] found that hypoxia downregulated the expression of Bcl-2 family pro-apoptotic proteins (Bid, Bad, and Bax) and inhibited apoptosis of tumor cells. Similarly, our experimental results showed that MDA-MB-231 cell apoptosis was significantly reduced in the hypoxic group compared with the control group, thus verifying the inhibitory effect of hypoxia on tumor cell apoptosis.

Self-renewal is a fundamental attribute of CSCs and has been demonstrated in xenograft animal experiments and colony-formation assays. Colony-formation assays reveal cells with the proliferative activity to form clones, such that the colony-formation rate reflects the proportion of cells with stemness. In the present study, we showed that hypoxia significantly increased the colony-formation rate and therefore the proportion of cells with stemness properties. We also measured hypoxia-induced changes in the percentage of BCSCs in MDA-MB-231 cells using flow cytometry analysis. Our results further confirmed the ability of hypoxia to increase the percentage of BCSCs in MDA-MB-231 cells and facilitate stemness transformation.

Hypoxia is a major feature of solid tumors and exerts multiple effects on the biological behavior of tumor cells. Our experiments showed that hypoxia had no significant cytotoxic effect, but did inhibit apoptosis and regulate stemness in cancer cells. Although these results further verified the multiple effects of hypoxia on cancer cells, the molecular mechanisms responsible for stemness regulation by hypoxia through dedifferentiation of differentiated cancer cells remain to be elucidated.
